# Numerical simulations help revealing the dynamics underneath the clouds of Jupiter

**DOI:** 10.1038/s41467-020-16680-0

**Published:** 2020-06-08

**Authors:** Johannes Wicht, Thomas Gastine

**Affiliations:** 1 Max Planck Institute for Solar System Research, Göttingen, Germany; 2 Institut de Physique du Globe de Paris, Paris, France

**Keywords:** Astronomy and astrophysics, Giant planets

## Abstract

Since its arrival at Jupiter in 2016, NASA’s Juno spacecraft has been performing high-precision measurement of the gravity and magnetic fields. When combined with numerical simulations, they provide a unique window to the dynamics in the planet’s deep atmosphere.

## A very different atmosphere

**Fig. 1:**
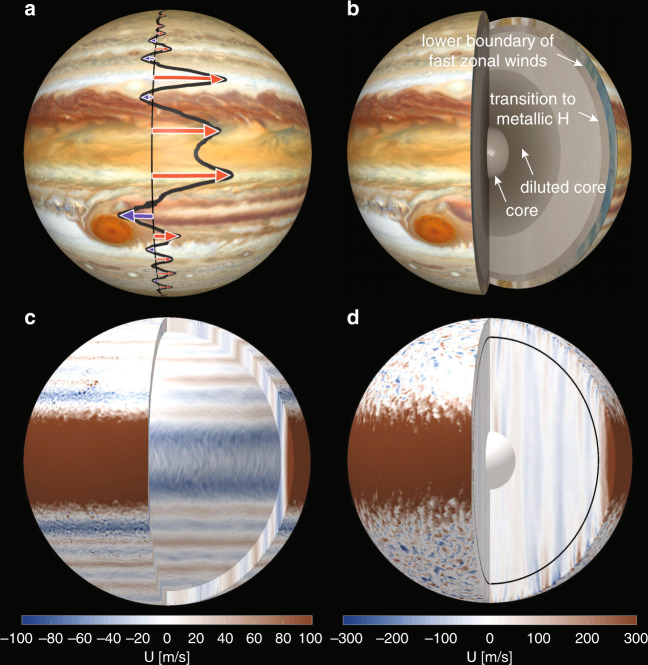
The zonal winds and interior structure of Jupiter. **a** Jupiter’s cloud level zonal jets projected onto a Hubble surface image. The black line shows the jet velocity profile while red arrows indicate eastward and blue arrows westward jet maxima. The fastest jets are eastward and reach a maximum velocity of about 130 m/s. **b** Interior model with an outer region of fast zonal jets (5% in radius), the transition to the metallic hydrogen region (10% in radius), a core (20% in radius), and a possible region of denser material that may consist of diluted core material^18^. **c** Color contours of the azimuthal flow *U* in a snapshot of a simulation in a shell covering 10% in radius^6^. Red and blue colors indicate eastward and westward winds respectively. **d** Color contours of the azimuthal flow *U* in a snapshot of a Jupiter-like dynamo simulation^10^. The black circle separates the weakly conducting outer and highly conducting inner region. Lorentz forces confine the dominant prograde equatorial jet to the outer region and brake the other jets. On time average, only the equatorial jet persists. The other azimuthal flows are more time dependent and merge to form a broad slow westward flow. The image of Jupiter’s surface in panels (**a**) and (**b**) is courtesy to NASA and ESA. Credits go to A. Simon (Goddard Space Flight Center) and M.H. Wong (University of California, Berkeley). The simulations illustrated in panels (**c**) and (**d**) have been performed with the dimensionless computer code MagIC. The code calculates the azimuthal flows in units of a Rossby number: Ro = *U/(*r_J_Ω). This has been rescaled to m/s by assuming a mean radius of r_J _ = 6.9911 × 10^6^ m and a planetary rotation rate of Ω = 1.759 × 10^−4^ s^−1^.

Earth’s atmosphere adds only a mere 20 km to the mean 6371 km radius of our rocky home planet. We all experience the complicated dynamics of this thin envelope when trying to keep pace with the capricious weather. Meteorologists rely on a continuous stream of data and enormous computational efforts to model this dynamics and have learned that Earth’s irregular surface and the variable solar irradiation play key roles. This is very different on Jupiter. The king among planets is a gas giant that mainly consists of hydrogen and helium. A denser core of unknown composition only occupies a smaller central portion (Fig. 1b). Since the typical atmospheric pressure reaches one bar at Earth’s surface, scientist traditionally also refer to the one bar level at a mean radius of r_J_ = 69,911 km as the lower boundary of Jupiter’s atmosphere. However, this is an arbitrary choice. Compared to the precious gas envelope around Earth, Jupiter’s hydrogen/helium envelope is virtually bottomless, and the solar radiation only penetrates a tiny outer fraction. Not surprisingly, the dynamics is also very different.

Unfortunately, we can only observe Jupiter’s top layers directly^1^. The wandering of the colorful clouds reveals a system of fast westward- and eastward-directed zonal jets that encircle the whole planet. The jets sculpture the planet’s beautiful stripes. The big red spot and numerous smaller spots, on the other hand, correlate with slower eddies. Earth-bound telescopes penetrate the thick cloud cover down to roughly 60 km below r_J_. The Galileo probe^2^, which entered Jupiter in 1995, survived to a depth of about 160 km. NASA’s Juno spacecraft^3^, currently in orbit around Jupiter, carries a powerful microwave radiometer, which can penetrate to a depth of perhaps 500 km. This is still less than 1% of the planet’s radius. In order to peer deeper, we must resort to more indirect means and combine observations with theoretical and numerical studies of the planet’s interior dynamics.

## Numerical simulations of jets and magnetic field

Studies of motions in deeper atmospheres that are driven by the cooling of the planet rather than by solar irradiation seem particularly relevant^4–6^. The respective results highlight the key role of the planetary rotation in structuring the turbulent flows. Three important jet properties become apparent: First, the jets naturally develop from the small-scale turbulence. Second, they form concentric cylinders that are aligned with the planetary rotation axis (Fig. 1c). Third, the width of the jets depends on the depth of the modeled shell and is most realistic for a lower boundary at 0.94 r_J_^5^. Which physical effect could put on the brakes at this specific depth? Perhaps the magnetic forces, which were neglected in these simulations?

Like Earth, Jupiter has a global magnetic field that is produced by a dynamo process. Earth’s dynamo operates in the liquid iron core, but what about Jupiter? Below about 0.9 r_J_, the extreme pressures squeeze the atoms so close together that hydrogen becomes metallic^7^. Above 0.9 r_J_, the electrical conductivity decreases rapidly in the so-called transition region and eventually becomes negligible. Since dynamo action and the magnetic Lorentz forces scale with conductivity, they are most efficiently in the inner metallic region but can be significant in the transition region. Where the Lorentz forces remain negligible in the very-outer atmosphere, the jets should retain their cylindrical geometry. Somewhere in the transition region, however, the Lorentz forces would kick in abruptly and quench the jets over a depth range of about 1000 km^8^.

To test these ideas, scientists ran a number of ambitious numerical simulations that not only model the jets but also the deeper dynamo processes. The results reveal interesting but also puzzling new aspects^9–12^. The magnetic field of Jupiter, like the field of Earth, is dominated by an axial dipole. However, the jets tend to promote magnetic field configurations with rather weak axial dipole contributions. The key to reproducing Jupiter’s magnetic field therefore lies in limiting the role of the jets in the dynamo process. Rather than simply quenching the jets in the transition region, however, the simulation choses another option (Fig. 1d): the Lorentz forces push the eastward equatorial jet to the outer envelope but slow down rather than truncate the remaining jets^10,11^.

**Fig. 2:**
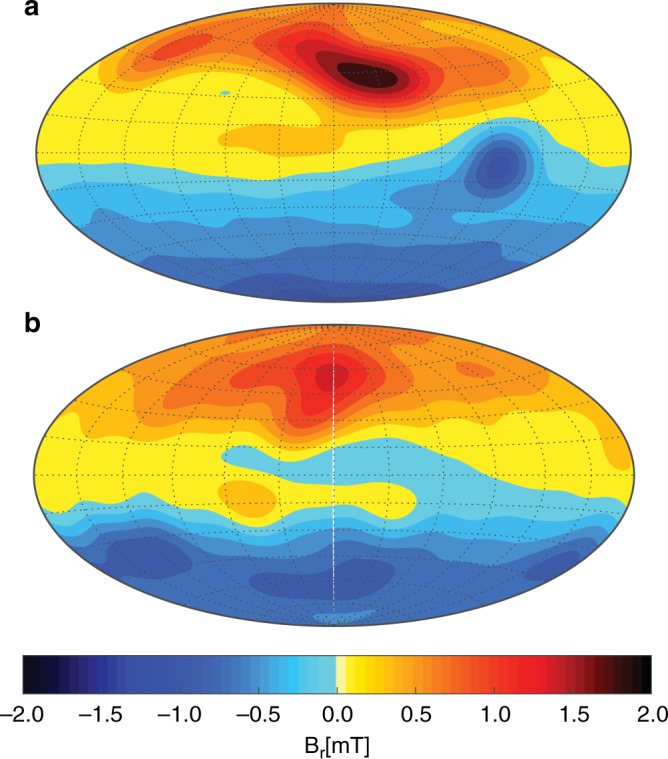
The magnetic field of Jupiter. **a** Jupiter's radial surface field model JRM09 based on Juno measurements^3^. **b** Snapshot of the radial surface field in a Jupiter-like dynamo simulations^10^. Outgoing field is shown in yellow and red and ingoing field in blue.

These simulations cannot exactly reproduce Jupiter’s complex jet structure (Fig. 1d). They also use unrealistically high viscosities to suppress the smallest turbulent features that cannot be resolved with current computer resources. One consequence is that the zonal winds are not as dominant as on Jupiter. However, the models seem to capture the essence of the dynamo process and reproduce the very inhomogeneous field distribution that sets the magnetic fields of Jupiter and Earth apart^13^. Particularly obvious is the concentration of outward directed field (Fig. 2a, yellow and red) in a latitudinal band between 30 and 60° north. In the southern hemisphere, a prominent patch of inward (blue) field sits just south of the equator, but overall the field seems more homogeneous than in the north. The simulation snapshot (Fig. 2b) shows very similar banded structures in the northern hemisphere^10^. The southern blue patch is missing, but similar features appear later in the simulation.

The numerical models generate the Jupiter-like magnetic field in a two-stage process. A deeper primary dynamo produces the main dipole-dominated magnetic field in the metallic region. A shallower secondary dynamo operates where the equatorial jet reaches sizeable conductivities in the transition region^10,11^ and generates the banded or patchy features observed at low to mid latitude. This suggests that the jets contribute to shaping the magnetic field of Jupiter. They also give rise to density difference and thus to tiny modifications of Jupiter’s gravity field. Both magnetic and gravity measurements can thus potentially constrain the deeper jet structure.

## Exploiting gravity and magnetic data

For the first time, Juno gravity measurements are precise enough to detect the tiny signature of the deeper zonal jets. However, the interpretation is difficult. The first attempts in principle adopt the cylindrical jet geometry but report that an additional gradual decay with depth is required^14,15^: While the jets blow with up to 150 m/s at cloud level, they slow down to about 10 m/s at 0.96 r_J_ and to 2 m/s at 0.94 r_J_. This is at odds with the abrupt decay expected from Lorentz forces. A comparison of the magnetic fields measured by Juno and by previous spacecrafts gives rise to further doubts: The modest variations over the 45-year time span covered by the missions indicate that the jet velocity cannot exceed 0.01 m/s at 0.94 r_J_^16^. Further analysis of gravity and magnetic data seem in order to resolve the contradictions.

## A stable layer in the outer atmosphere of Jupiter?

A recent numerical study^17^ of jets in a simplified Cartesian box puts a promising new idea on the table: An additional stable layer where the density stratification tend to suppresses radial motions. Lorentz forces drive radial flows in the transition region, which are  much slower than the jets and therefore normally have little effect. However, once the radial flows penetrate a stable layer and encounter the density stratification, they can promote a very effectively quenching of the jets.

The upper boundary of the stable layer should lie between 0.94 r_J_ and 0.96 r_J_. Interestingly, some newer interior structure models suggest a somewhat deeper stable layer below 0.93 r_J_^18^. However, it remains unclear which physical mechanism could support such a layer. Helium rain, a process deemed responsible for a thick stable layer in Saturn, only happens in metallic hydrogen and thus not above 0.9 r_J_ in Jupiter.

## Conclusion

The combination of observations and numerical simulations provides a powerful tool for peering underneath the cloud deck of Jupiter. The results suggest that the fast jets are limited to the outer 4–6% in radius, assume a cylindrical geometry, and are abruptly quenched at the lower boundary, perhaps by a combination Lorentz forces and a stable layer. While first evaluations of Juno gravity data propose a more gradual quenching, the analysis is complex and ambiguous and leaves room for alternative models.

State-of-the-art numerical dynamo simulations reproduce many features of Jupiter’s magnetic field and highlight the important role of the dominant equatorial jet. However, they fail in reproducing the multiple jet system. Two options for improvement are the adoption of lower viscosities and the implementation of a stable layer. Juno gravity data also indicate a higher concentration of heavy elements below perhaps 0.6 r_J_^18^. Future numerical studies could clarify whether such a scenario is consistent with Jupiter’s magnetic field.

The simulations also suggest that Jupiter's magnetic field bears signs of zonal jet action. However, deducing the jet properties from the magnetic field structure is very challenging. Observations of magnetic field variations, on the other hand, are an established tool for accessing the dynamic in Earth’s core and have already proven useful at Jupiter^16^. There is a chance that Juno will observe minor variations during its mission time. More promising data will become available when ESA’s Juice spacecraft arrives at Jupiter in 2029.

## Data Availability

Most of the simulations discussed here were performed with the code MagIC, which is freely available at GitHub: https://github.com/magic-sph.
